# Why equal treatment is not always equitable: the impact of existing ethnic health inequalities in cost-effectiveness modeling

**DOI:** 10.1186/1478-7954-12-15

**Published:** 2014-06-02

**Authors:** Melissa McLeod, Tony Blakely, Giorgi Kvizhinadze, Ricci Harris

**Affiliations:** 1 Department of Public Health, University of Otago Wellington, PO Box 7343 23 Mein Street, Newtown Wellington, New Zealand

**Keywords:** Heterogeneity, Cost effectiveness, Equity, Māori, New Zealand, Ethnicity

## Abstract

**Background:**

A critical first step toward incorporating equity into cost-effectiveness analyses is to appropriately model interventions by population subgroups. In this paper we use a standardized treatment intervention to examine the impact of using ethnic-specific (Māori and non-Māori) data in cost-utility analyses for three cancers.

**Methods:**

We estimate gains in health-adjusted life years (HALYs) for a simple intervention (20% reduction in excess cancer mortality) for lung, female breast, and colon cancers, using Markov modeling. Base models include ethnic-specific cancer incidence with other parameters either turned off or set to non-Māori levels for both groups. Subsequent models add ethnic-specific cancer survival, morbidity, and life expectancy. Costs include intervention and downstream health system costs.

**Results:**

For the three cancers, including existing inequalities in background parameters (population mortality and comorbidities) for Māori attributes less value to a year of life saved compared to non-Māori and lowers the relative health gains for Māori. In contrast, ethnic inequalities in cancer parameters have less predictable effects. Despite Māori having higher excess mortality from all three cancers, modeled health gains for Māori were less from the lung cancer intervention than for non-Māori but higher for the breast and colon interventions.

**Conclusions:**

Cost-effectiveness modeling is a useful tool in the prioritization of health services. But there are important (and sometimes counterintuitive) implications of including ethnic-specific background and disease parameters. In order to avoid perpetuating existing ethnic inequalities in health, such analyses should be undertaken with care.

## Background

Traditional cost-effectiveness approaches place an equal value on increases in health regardless of whom they are accruing to, albeit discounted over time, despite there being international evidence that people are willing to sacrifice some total population health gain in order to improve the health of certain groups [1-3] (e.g., younger age, severe disease). A range of methods to incorporate equity concerns into cost-effectiveness analyses have been developed [4-6] but are rarely applied [7], even though equity is a fundamental goal of New Zealand (NZ) and other health systems [8,9].

A critical first step toward incorporating equity into cost-effectiveness analyses is to appropriately model interventions by population subgroups. The current recommendation from the National Institute for Health and Clinical Excellence (NICE) is that subpopulation analyses should occur where there is “an a priori expectation of differential clinical or cost effectiveness due to known, biologically plausible mechanisms, social characteristics or other clearly justified factors” [10].

The relative cost-effectiveness of an intervention across population groups is influenced both by a) differences in specifications of the intervention itself (such as coverage, cost, or effectiveness), and b) from using group-specific epidemiological parameters such as disease incidence, mortality, and morbidity rates. In this paper we aim to illustrate how health gain variations by social group are influenced by differences in these latter underlying epidemiological parameters, as a fundamental first step toward an equity agenda.

This work was undertaken within a cancer research program and uses as our case example a hypothetical cancer intervention in NZ, focusing on between-ethnic group heterogeneity. There are significant inequalities in cancer incidence [11] and outcomes [12], life expectancy [13], and the total burden of disease for the indigenous Māori population when compared to the non-Māori population. We select three cancers as case studies (lung, female breast, and colon cancer) that have variations in incidence rate differences by ethnic group as well as overall survival. Lung cancer incidence is two to three times higher for Māori [11,14] and mortality is three to four times higher for Māori than non-Māori [15]. The breast cancer incidence rate among Māori women (aged over 45 years) is 17% higher than in non-Māori women [11,14], and mortality rates are about 70% higher [15]. Colorectal cancer is diagnosed at a 40% lower rate in Māori than in non-Māori [11,14], but Māori are often diagnosed at a later stage of disease, and as a result, Māori survival is worse than non-Māori [15].

For each cancer, we model a hypothetical treatment that reduces the excess mortality due to the cancer by 20% for both Māori and non-Māori. We include a fixed intervention cost ($2,500 per month in the treatment phase) and calculate cost offsets due to patients living longer (which increases health system costs) as well as not dying (which decreases costs). The main focus of this paper is how health-adjusted life years (HALYs) (and to a lesser degree, incremental cost-effectiveness ratios (ICERs)) vary by ethnic group as one sequentially specifies differences in cancer mortality, background mortality, and expected background morbidity.

## Methods

Study methods followed the Burden of Disease Epidemiology, Equity and Cost-Effectiveness programme (BODE^3^) Protocol and applied a health system perspective [16]. Results are presented as expected values for average levels of input parameters to allow for an easy comparison between Māori and non-Māori for three selected age groups (50–54 years, 65–69 years, and 80–84 years).

### Model structure

**Figure 1:**
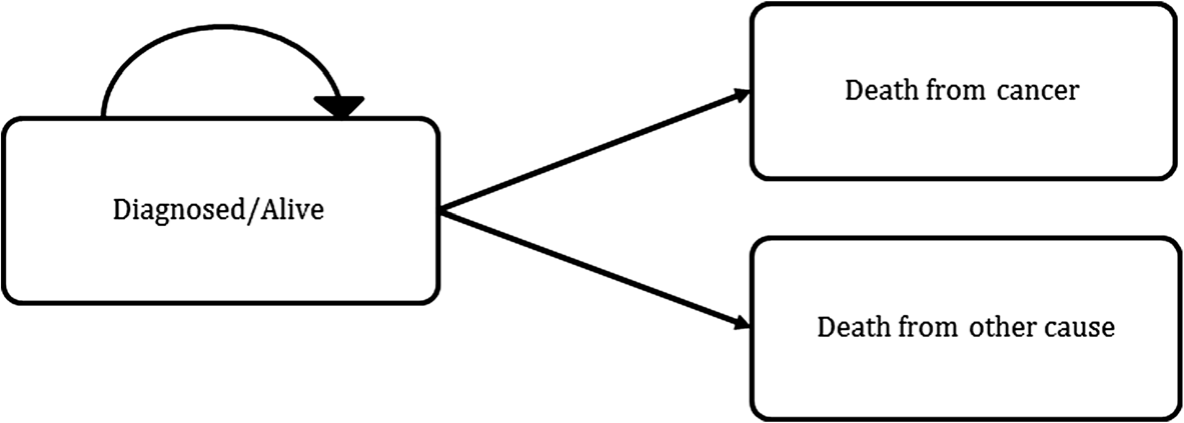
Stylized depiction of Markov Model.

A three-state Markov Model was developed with a monthly cycle length and the same structure for each cancer (Figure 1). Excess mortality from the cancer of interest and the population background mortality rates were used to calculate transition probabilities to death from cancer or death from other causes, respectively. These parameters varied by socio-demographic group and time. For all three cancers, the HALYs and costs were calculated based on the absorbing state (i.e., death from cancer in question or other causes) and the time since diagnosis that this state was reached. Key parameters included disability weights and health system costs by disease phase; an initial period of diagnosis and treatment, periods of pre-terminal and terminal disease, a phase of remission occurring in between, and (for survivors) background population expectations. For those who died (from cancer or other causes), we retrospectively constructed their disease trajectory to include increased morbidity and costs in the final months of life. The length of each phase, and the period of time a survivor remained in the remission phase before becoming statistically cured varied across the three cancers and by time of death (Table 1). The time horizon for the full model was from diagnosis until 110 years of age.

**Table 1:** Phase duration and disability weights for lung, female breast, and colon cancer

	**Lung cancer**	**Female breast cancer**	**Colon cancer**
**Phase duration (months)**			
Diagnosis and treatment	5	6	9
Pre-terminal	5	11	3
Terminal duration	1	1	1
Statistical cure time	72	240	96
Disability weights*			
Diagnosis and treatment	0.469	0.194	0.288
Pre-terminal	0.539	0.512	0.539
Terminal	0.548	0.520	0.548
Remission**	0.315	0.174	0.167

*Disability weights were sourced from the 2010 Global Burden of Disease [17] with modification to the NZ distribution of cancers [16].

**Remission refers to the period between diagnosis and treatment and either pre-terminal disease or statistical cure.

Note: For example, an individual dying of lung cancer one year after diagnosis will spend 5 months in the treatment and diagnosis phase; 1 month in remission; 5 months in the pre-terminal phase, and their final month in the terminal phase.

### Input parameters

#### ***Ethnicity categories***

Individuals were classified into mutually exclusive groups of Māori and non-Māori within each dataset.

#### ***Incidence rates***

Incidence rates for lung cancer, female breast cancer, and colon cancer for 2006 disaggregated by age group (45–49, 50–54,….84–89, 90–94 years), sex, ethnicity (Māori and non-Māori), and area deprivation (NZDep2006 1–3, 4–6, 7–10) were sourced from NZ Cancer Registry data merged with linked census-cancer data to specify ethnic and deprivation variations in incidence [18].

#### ***Excess and background mortality rates***

Excess mortality rates (deaths attributable) to lung cancer, female breast cancer, and colon cancer differentiated by age, sex, ethnicity, and deprivation were derived from the NZ Cancer Registry using relative survival [19,20] (methods described elsewhere [21]). Background mortality rates (all causes) by socio-demographic strata were derived from standard NZ life tables and projected to the future with different annual percentage declines (up to 2026) in mortality rates for Māori (2.25%) and non-Māori (1.75%) to match improvements in life expectancy over the last 100 years. In models where Māori background mortality was set at the level of non-Māori, the annual percentage change to 2026 was also set to the non-Māori level of 1.75%. Sensitivity analyses of 0-4% reductions were undertaken and had little impact on the ICER (results not shown).

#### ***Expected background morbidity***

**Figure 2:**
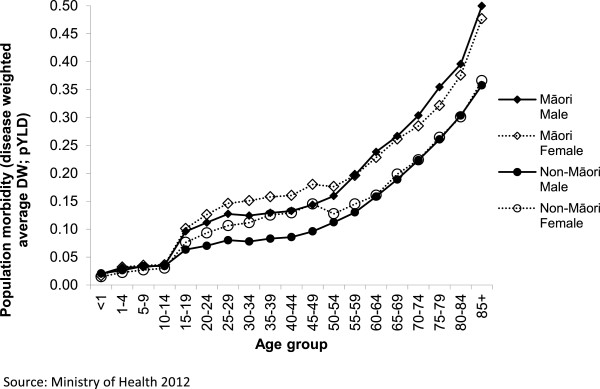
**Population morbidity by ethnicity, gender, and age group.** Source: NZ Ministry of Health 2013 [22].

Expected background morbidity uses the average ethnic and age-specific prevalent years of life lived in disability from the New Zealand Burden of Disease Study [22]. This limits the maximum HALYs that can be gained both with increasing age and for Māori compared to non-Māori (Figure 2). For example, a year of life saved for a non-Māori female aged 70–74 years of age has a maximum theoretical HALY gain of (1-pYLD) = 1 – 0.224 = 0.776.

### The intervention

The direct costs of the three cancer interventions were set to $2,500 per month for the diagnosis and treatment phase, a value that produced ICERs around the funding threshold of $40,000 for the middle age group. The intervention effect for all three cancers was a 20% reduction in monthly excess mortality. A 3% per annum discount rate was applied to costs and benefits.

#### ***Intervention benefits***

HALYs calculated here are similar to quality-adjusted life years (QALYs), except in calculating morbidity: we set a maximum potential envelope of health gain allowing for background population morbidity and we use disability weights to account for morbidity from the cancer itself. Health benefits are presented as both baseline (from current health services) and incremental (those attributable to the intervention) HALYs per case (calculated as the baseline or incremental HALYs divided by the number of cancer cases) and per 100,000 ethnic-specific population (calculated as the incremental HALYs divided by the 2006 census population for Māori or non-Māori). The relative risks of HALY gains were calculated by dividing the Māori incremental HALY gains by the non-Māori. The ICER is calculated as the net change in costs divided by net health gain.

#### ***Health system costs***

The average health system costs for the NZ population were calculated using the average of all health system use and attendant costs for each age group by analyzing total NZ population health data linked with 2011 costing using gamma regression. Additional health system costs for patients with each of the three cancers were estimated by disease phase. We separately determined expected costs for those in the last six months of life for those dying from causes other than cancer (see Additional file 1).

### Analyses/modeling

Five models were run for each cancer in order to identify the absolute and relative impacts of varying input parameters by ethnicity. Each subsequent model added in another layer of epidemiological heterogeneity (Table 2). Sensitivity analyses of direct cost and effect size were undertaken for each cancer.

**Table 2:** Model parameter specifications

	**Model 1**	**Model 2**	**Model 3**	**Model 4**	**Model 5**
Cancer incidence	**Ethnic-specific**	Ethnic specific	Ethnic specific	Ethnic specific	Ethnic specific
Cancer excess mortality	Non-Māori	**Ethnic-specific**	Ethnic specific	Ethnic specific	Ethnic specific
Disability weights	Off	Off	**On**	On	On
Background mortality rate	Non-Māori	Non-Māori	Non-Māori	**Ethnic-specific**	Ethnic specific
Prevalent years lost to disability	Off	Off	Off	Off	**Ethnic-specific**

The significance of the bold text is to identify how the model differs from the preceding model.

## Results

### Health or HALYs gained per case

#### ***Lung cancer***

The modeled incremental health gains per case for the lung cancer intervention are greater for non-Māori compared to Māori across all five models and three age groups (Table 3 and Figure 2). As expected, the gain in life years decreases with increasing age (Table 3). Males had higher ICERs than females, with similar absolute and relative differences between Māori and non-Māori males and females (Additional file 2: Figure S1).

**Table 3:** Modeled lung, breast, and colon cancer interventions baseline and incremental benefits, incremental costs, and incremental cost-effectiveness, for Māori and non-Māori aged 50–54, 65–69, and 80–84 years, with varying input parameters (Models 1–5)

	**50-54 years**					**65-69 years**					**80-84 years**				
	**HALY per 100,000 pop^**	**Baseline HALY per case**	**Inc HALY per case**	**HALY per case% change**	**ICER**	**HALY per 100,000 pop^**	**Baseline HALY per case**	**Inc HALY per case**	**HALY per case% change**	**ICER**	**HALY per 100,000 pop^**	**Baseline HALY per case**	**Inc HALY per case**	**HALY per case% change**	**ICER**
**Lung cancer**															
**Non-Māori**															
Model 1/2	41	4.13	1.49	0	13200	126	2.21	0.89	0	20000	98	1.01	0.37	0	40900
Model 3/4	39	3.64	1.41	-5.4	14000	116	1.80	0.81	-9.0	21800	82	0.69	0.31	-16.2	49100
Model 5	31	2.94	1.13	-24.2	17500	85	1.35	0.60	-32.6	29600	54	0.46	0.20	-45.9	74300
**Māori**															
Model 1	145	4.02	1.47	0	13300	419	2.14	0.87	0	20200	230	1.00	0.37	0	40700
Model 2	127	2.90	1.29	-12.2	14600	346	1.52	0.72	-17.2	23000	185	0.74	0.30	-18.9	47600
Model 3	119	2.49	1.21	-17.7	15500	312	1.18	0.65	-25.3	25400	149	0.47	0.24	-35.1	59000
Model 4	105	2.25	1.07	-27.2	16900	260	1.03	0.54	-37.9	29500	126	0.43	0.20	-45.9	68200
Model 5	78	1.69	0.79	-46.3	22900	174	0.71	0.36	-58.6	44000	71	0.26	0.11	-70.3	120700
**Breast cancer**															
**Non-Māori**															
Model 1/2	65	17.53	0.74	0	24500	82	11.62	0.61	0	30000	40	6.07	0.25	0	66800
Model 3/4	64	16.80	0.73	-1.4	24800	81	10.92	0.60	-1.6	30500	39	5.49	0.24	-4.0	68900
Model 5	50	13.37	0.57	-23.0	31900	58	8.05	0.43	-29.5	42600	25	3.62	0.16	-36.0	107300
**Māori**															
Model 1	109	17.26	0.75	0	24300	138	11.42	0.61	0	29800	68	6.04	0.25	0	66400
Model 2	141	15.84	0.96	+28.0	19900	172	10.28	0.76	+24.6	25000	87	5.56	0.32	+28.0	53700
Model 3	139	15.12	0.95	+26.7	20100	169	9.61	0.75	+23.0	25400	84	5.01	0.31	+24.0	55800
Model 4	122	13.63	0.83	+10.7	22400	135	8.17	0.60	-1.6	30600	66	4.28	0.24	-4.0	68600
Model 5	88	10.14	0.60	-20.0	31100	88	5.55	0.39	-36.1	47000	36	2.43	0.13	-48.0	126300
**Colon Cancer**															
**Non-Māori**															
Model 1/2	36	13.05	1.28	0	15300	159	8.95	0.85	0	22200	159	4.31	0.42	0	41400
Model 3/4	35	12.44	1.25	-2.3	15600	154	8.36	0.82	-3.5	22900	149	3.82	0.4	-4.8	44100
Model 5	28	9.99	1	-28	19600	112	6.19	0.6	-29.4	31300	98	2.52	0.26	-38.1	67400
**Māori**															
Model 1	27	12.71	1.27	0	15300	114	8.64	0.83	0	22500	109	4.21	0.42	0	41700
Model 2	33	9.83	1.53	20.5	13300	138	6.75	1.01	21.7	19400	130	3.28	0.5	19	35900
Model 3	32	9.29	1.5	18.1	13600	133	6.23	0.97	16.9	20100	122	2.85	0.47	11.9	38400
Model 4	28	8.31	1.32	2.4	14900	110	5.27	0.8	-3.6	23400	100	2.42	0.38	-9.5	45700
Model 5	21	6.18	0.97	-23.6	20300	72	3.53	0.53	-36.1	35500	54	1.36	0.21	-50	83900

Model 1 – Ethnic-specific cancer incidence, non-Māori cancer excess mortality, and background mortality rates for all groups, no disability weights for cancer phases, no background population morbidity.

Model 2 – Same as Model 1, with ethnic-specific cancer excess mortality rate.

Model 3 – Same as Model 2, with disability weights for cancer phases.

Model 4 – Same as Model 3, with ethnic-specific background mortality rates.

Model 5 Heterogeneity model – Same as Model 4, with ethnic-specific background population morbidity.

^per 100,000 ethnic-specific resident population.

Note: The incremental cost per case for Model 1 was similar for Māori and non-Māori and reduced for Māori in Models 2 and 4 to be less than $2000 difference between Models 1–5.

The greatest health gains per case are measured in Model 1, which uses non-Māori excess mortality from lung cancer for both groups. The health gains in Models 1 and 2 are additional years of life, as the disability weights and prevalent years lost to disability have been turned off. In Model 1 of the lung cancer intervention, given current health services, non-Māori with lung cancer aged 65–69 years will live on average an additional 2.2 years, and Māori an additional 2.1 years (baseline HALYs).

The incremental life years gained through our hypothetical intervention (20% reduction in excess mortality) are 0.89 for non-Māori and 0.87 for Māori. This small difference results from including the deprivation distributions of the two populations in the base model (Māori are relatively more deprived), as incidence, cancer excess mortality, and background mortality vary by deprivation.

Model 2 resets the Māori excess mortality (i.e., survival) to the Māori values (Table 3). This reduces both the measured baseline and incremental life years gained per case for Māori across the three age groups, and results in a higher ICER for Māori of $23,000 compared to $20,000 for non-Māori.

Model 3 adds disability weights by phase of lung cancer. As a result, the HALYs gained are less than the life years measured in Models 1 and 2 for both Māori and non-Māori and across all age groups, and the ICERs for both groups increase. The absolute changes in HALYs per case are similar between ethnic groups, but the percentage reductions are greater for Māori due to the lower HALYs gained per case. Compared with Model 1, the health gain per case was reduced by 18-35% for Māori across the three age groups and by 5-16% for non-Māori (Table 3).

**Figure 3:**
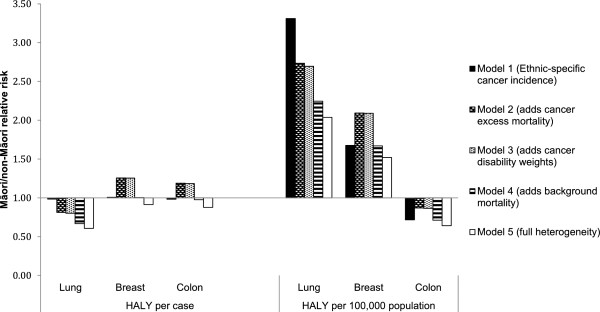
Māori/non-Māori incremental HALY gain relative risks, per case and per 100,000 ethnic-specific population.

Model 4 additionally includes ethnic-specific background mortality rates for Māori and non-Māori. Incorporating the lower life expectancy of Māori into Model 4 results in a large reduction in measured HALYs gained per case (27-45% reduction) (Table 3) and an increase in the ICER to $29,500.Model 5, full heterogeneity, includes ethnic-specific levels of prevalent morbidity for Māori and non-Māori. The lower life expectancy of Māori (Model 4) as well as a higher burden of comorbid illness limits the measured gains that can be achieved through an intervention more for Māori than for non-Māori. While the life years gained for Māori and non-Māori in Model 1 were very similar, in Model 5 the HALYs gained per case for Māori aged 65–69 years are now 0.61 of those for non-Māori of the same age, resulting in an ICER of $44,000 for Maori and $29,600 for non-Maori (Figure 3).

#### ***Breast and colon cancer***

Similar to the lung cancer Model 1, Māori in the female breast and colon cancer interventions have similar baseline and incremental life years gained per case compared to non-Māori when cancer incidence is the only variable that differs by ethnicity (Table 3). However, unlike the decrease in incremental gains per case seen with lung cancer, adding ethnic-specific variation in cancer excess mortality for these two diseases results in greater incremental gains per case for Māori relative to non-Māori (female breast cancer RR = 1.26, colon RR = 1.19) (Figure 3). Similar to the lung cancer intervention, adding in Māori background mortality (Model 3) and prevalent years lost to disability (Model 4) for the female breast and colon interventions reduces the baseline and incremental HALYs gained per case, with the impacts on the incremental HALYs being greater for Māori than for non-Māori (Figure 3).

### HALYs gained per 100,000 ethnic-specific population

HALYs gained on a population basis (per 100,000 ethnic-specific population) present a very different pattern to HALYs gained per case (Figure 3), due to the differences in incidence rates. For the lung and female breast cancer interventions in those aged 65–69 years, the gains per 100,000 population for Māori are greater than for non-Māori across all five models. These range from 3.31 to 2.04 times the population HALY gain for Māori for the lung cancer intervention (compared to non-Māori) and 2.09 to 1.52 times the gain for Māori (compared to non-Māori) with the female breast cancer intervention. The relative risk for Model 1 is equivalent to the incidence rate ratio for Māori compared to non-Māori, and therefore is the highest for lung cancer at RR = 3.31 (Māori have three times the lung cancer incidence of non-Māori) and lowest for colon cancer with a RR = 0.72.Adding ethnic-specific excess mortality reduces the modeled population-level benefit for Māori relative to non-Māori for the lung cancer intervention (from RR = 3.31 to RR = 2.73), but increases the relative benefit for Māori from the female breast and colon cancer interventions (Figure 3). For all three cancers, adding ethnic-specific background mortality and prevalent years lost to disability reduces the measured population level benefits for Māori relative to non-Māori.

Two-way sensitivity analyses varying direct costs and effect size have large impacts on the ICERs for all three cancer interventions (Additional file 3: Figure S2): increasing direct costs increased the ICER, and increased intervention effectiveness (large percentage decreases in excess mortality) decreased the ICER. Changing the direct cost and effect size of the intervention did not substantively alter the relative difference in ICERs between Māori and non-Māori.

## Discussion

The cost-effectiveness of interventions will often vary between population groups and therefore has the potential to play an important and practical role in decision making, when considering which groups to fund an intervention for, as well as allowing for comparisons of different interventions for a single subpopulation.

Two sets of parameters were examined in this paper: background parameters of mortality (and resultant expected remaining life expectancy) and morbidity and disease-specific parameters of cancer incidence and survival. For the three cancer interventions, including existing inequalities in background parameters (life expectancy and comorbidities) for Māori attributes less value to a year of life saved compared to non-Māori, lowers the relative health gains for Māori, and therefore increases the resulting cost-effectiveness ratio (making the intervention appear less cost-effective).

In contrast, ethnic inequalities in cancer parameters had less predictable effects. The intervention modeled in this paper worked by reducing the excess mortality rate of each of the three case study cancers by 20%. Therefore, ethnic differences in the excess mortality rates from the case study cancers had an important influence on the benefits of the intervention. Despite excess mortality rates for Māori being higher for all three case study cancers, per case HALYs gained for Māori from the lung cancer intervention were less than for non-Māori. This is because the absolute survival *proportions* for breast and colon cancer are higher than for lung cancer (i.e., not 10% or less), meaning that equivalent percentage reductions in the excess mortality *rate* can translate into larger absolute survival gains for a population (such as Māori) with higher baseline excess mortality (Additional file 4).From the perspective of reducing inequalities between population groups, there is value in examining both the HALYs gained per case and per population. Population gains are driven by the incidence of the disease, which is clearly demonstrated with the lung cancer model results (Figure 3). At a per case basis, the lung cancer intervention resulted in less measured HALYs gained per case for Māori; however, at the population level, the gains for Māori were two to three times that of non-Māori.

There are potential limitations to this work. The presentation of results as best estimates (without uncertainty analyses) is justified in that the intervention was hypothetical, and the purpose of the paper was to demonstrate the absolute and relative changes by ethnic groups. It is critical that “real” cost-effectiveness analyses include uncertainty. Where uncertainty in final health gain, costs, and cost-effectiveness is moderate to large, differences due to subpopulation heterogeneity may be lost in the cloud of parameter uncertainty. Nevertheless, a systematic difference between subpopulations persists beneath this uncertainty.

Within this study we modeled a hypothetical intervention that achieved equal coverage, effectiveness, and incurred the same direct costs by ethnicity. Sensitivity analyses of direct cost and effect size changed the ICERs for both groups but had little influence over the relative ICERs for Māori compared to non-Māori. Unfortunately for Māori, there is often an additional layer of disadvantage in cost-effectiveness analyses (as well as in health outcomes) resulting from health services achieving lower coverage or incurring higher costs than for non-Māori.

There is a need for further examination of the impact of heterogeneity for different intervention types, such as preventive interventions that change incidence and stage at presentation.

In cost-effectiveness analyses comparing ethnic/racial groups, it is common practice to use ethnic-specific background and disease parameters [23-25]. However, there is some debate in the literature around the fairness of using group-specific parameters (e.g., life expectancies [26]). One view is that unfair discrimination occurs by virtue of valuing one subpopulation less than another, regardless of the results of the analysis [27]. Holtgrave, in a paper on HIV prevention, recommends avoiding such “methodological discrimination” by using general population (as opposed to race/ethnic-specific) life expectancy, quality of life estimates, and care and treatment costs in cost-utility base case analyses, with sensitivity analyses around these parameters [28].

We suggest that there are two approaches to subpopulation cost-effectiveness modeling. First, there is the approach that seeks to model the health gains as they will occur, *given* background health inequalities. For example, to compare a range of interventions within the Māori population to select the intervention with the biggest health gain, you should use the parameters actually existing in the population. Second, there is an approach that seeks to compare subpopulations – here extreme care must be exercised. Our work, and that of Holtgrave before [28], demonstrates that naïve comparisons between subpopulations by incorporating a shorter life expectancy and higher burden of morbidity for one group (which are themselves the results of discrimination) is unjust. We recommend in this situation that researchers should include a standard life expectancy and background morbidity for both groups, with sensitivity analyses around the background and disease-specific parameters.

Cost-effectiveness analyses are an important and useful tool for health service prioritization. However, users should understand and explicitly consider how current methods may impact equity for priority groups within populations.

## Competing interests

The author(s) declare that they have no competing interests.

## Authors’ contributions

MM was involved in all aspects of the study and drafted the manuscript. TB participated in study design, data analysis and interpretation, and coordination and helped to draft the manuscript. GK participated in the design of the study and performed the statistical analysis. RH participated in the interpretation of the results and assisted with drafting the manuscript. All authors read and approved the final manuscript.

## Supplementary Material

Additional file 1 Average health system costs ($NZ) for total New Zealand population, and health system costs for the last six months of life, by age and gender, 2011. Click here for file

Additional file 2: Figure S1 Modeled lung cancer intervention ICERs by age, gender, and ethnicity. Click here for file

Additional file 3: Figure S2 Sensitivity analyses of direct cost and intervention effect size. Click here for file

Additional file 4 Further explanation for differences in relative survival proportions. Click here for file
