# Supplementary material for: Why equal treatment is not always equitable: the impact of existing ethnic health inequalities in cost-effectiveness modeling
Source: Popul Health Metr. 2014 Jun 2;12:15. doi: 10.1186/1478-7954-12-15 (PMC4047777; doi:10.1186/1478-7954-12-15)
Supplement: Additional file 1 — Average health system costs ($NZ) for total New Zealand population, and health system costs for the last six months of life, by age and gender, 2011. [file 1478-7954-12-15-S1.pdf]

Appendix 1: Health system costs

Table 6 Average health system costs ($NZ) for total New Zealand population, and health system costs for the last six months of life, by age and gender, 2011

Age group (yrs)	Male		Female	
	Average monthly cost	Last 6 months of life	Average monthly cost	Last 6 months of life
0	132.58	1655.37	149.24	2004.90
1-4	115.14	1536.45	129.60	1860.86
5-9	86.70	1309.57	97.59	1586.08
10-14	71.53	1157.04	80.51	1401.35
15-19	64.05	1056.95	72.10	1280.12
20-24	61.70	995.68	69.45	1205.91
25-29	63.33	964.76	71.29	1168.46
30-34	68.64	959.01	77.26	1161.50
35-39	77.84	975.46	87.62	1181.43
40-44	91.50	1012.63	103.00	1226.45
45-49	110.48	1070.10	124.36	1296.05
50-54	135.77	1148.15	152.82	1390.58
55-59	168.23	1247.54	189.37	1510.96
60-64	208.28	1369.18	234.45	1658.28
65-69	255.28	1513.89	287.35	1833.55
70-74	306.91	1682.02	345.46	2037.17
75-79	358.61	1873.01	403.66	2268.49
80-84	403.51	2084.96	454.20	2525.20
85-89	433.21	2314.09	487.63	2802.71
90+	439.71	2554.23	494.95	3093.55
