# Supplementary material for: Why equal treatment is not always equitable: the impact of existing ethnic health inequalities in cost-effectiveness modeling
Source: Popul Health Metr. 2014 Jun 2;12:15. doi: 10.1186/1478-7954-12-15 (PMC4047777; doi:10.1186/1478-7954-12-15)
Supplement: Additional file 4 — Further explanation for differences in relative survival proportions. [file 1478-7954-12-15-S4.pdf]

Appendix 2: Differences in relative survival proportion

For a given annual (or other unit time) excess mortality rate (EMR) from cancer, the relative survival proportion is given by:

1 – exp(-EMR)

For example, if the EMR=0.1 per person year, then the one-year relative survival proportion is 0.905. The relative survival proportion is that among people surviving from other competing causes of death. If the EMR is 1.0 per person year, the relative survival is 0.368.

Assume the above two examples are for non-Māori, for cancer A (good survival) and cancer B (poor survival,) respectively, for baseline or business as usual (i.e. without any treatment). Next, assume that Māori have a 50% higher EMR than non-Māori, or an excess mortality rate ratio (EMRR) of 1.5. Then the Māori relative survival for cancer A is exp(-0.15) = 0.861, and for cancer B is exp(-1.5) = 0.223.

Finally, assume that the treatment effect is a 20% reduction in the EMR applied similarly to each cancer and each ethnic group. Then, the relative survival post-treatment for cancer A will be:

- Exp(-0.08) = 0.923 for non-Māori, a gain in relative survival of 0.923 – 0.905 = 0.018.
- Exp(-0.12) = 0.887 for Māori, a gain in relative survival of 0.887 – 0.861 = 0.026.
- Resulting in a 0.026 – 0.018 = 0.008 greater survival gain for Māori.

And the relative survival post-treatment for cancer B will be:

- Exp(-0.8) = 0.449 for non-Māori, a gain in relative survival of 0.449 – 0.368 = 0.081.
- Exp(-1.2) = 0.301 for Māori, a gain in relative survival of 0.301 – 0.223 = 0.078.
- Resulting in a 0.081 – 0.078 = -0.003 difference in survival gains between Maori and non-Māori, i.e. a lesser survival gain for Māori.

Thus, and perhaps counter-intuitively, the same percentage reductions in the EMR can (when the baseline EMR is particularly high) result in less absolute survival gain for Māori despite Māori having higher EMRs. This is due to high EMRs resulting in very poor survival chances (i.e. survival proportion tending towards zero, and bounded by zero), with little absolute gains in survival possible.

The figures below show the difference in relative survival proportions between Māori and non-Māori on the y-axis (greater than zero being a greater gain for Māori) for:

- Four treatment effects: 5%, 10%, 20% and 40% reductions in the EMR.

- Varying amounts of inequality between Māori and non-Māori in the EMR parameterized as an excess mortality rate ratio on the x-axes, ranging from 0.4 (i.e. 60% lower EMR for Māori compared to non-Māori) to 3.0 (i.e. 200% higher EMR for Māori).
- And varying non-Māori baseline (i.e. pre-treatment) EMRs shown by each separate curve, ranging from 0.05 per person year to 2.0 per person-year.

Note that the y-axis scale varies in each figure, but the gridlines have been kept at 0.1 increments to aid visual comparison.

Focusing on the commonly experienced magnitude of survival (or more strictly speaking EMR) inequalities between Maori and non-Māori of between 1.1 to 1.5 EMRR inequalities, the figures demonstrate for high EMRs (e.g. greater than 1.0 per person year) that absolute survival gains will be less for Māori than non-Māori. This is what is happening with lung cancer in the main paper. Conversely, for cancers with lower EMRs and better survival (e.g. colorectal and breast cancer), survival gains are greater for Māori.
