# Supplementary material for: Why equal treatment is not always equitable: the impact of existing ethnic health inequalities in cost-effectiveness modeling
Source: Popul Health Metr. 2014 Jun 2;12:15. doi: 10.1186/1478-7954-12-15 (PMC4047777; doi:10.1186/1478-7954-12-15)
Supplement: Supplementary file 3 [file 1478-7954-12-15-S2.docx]

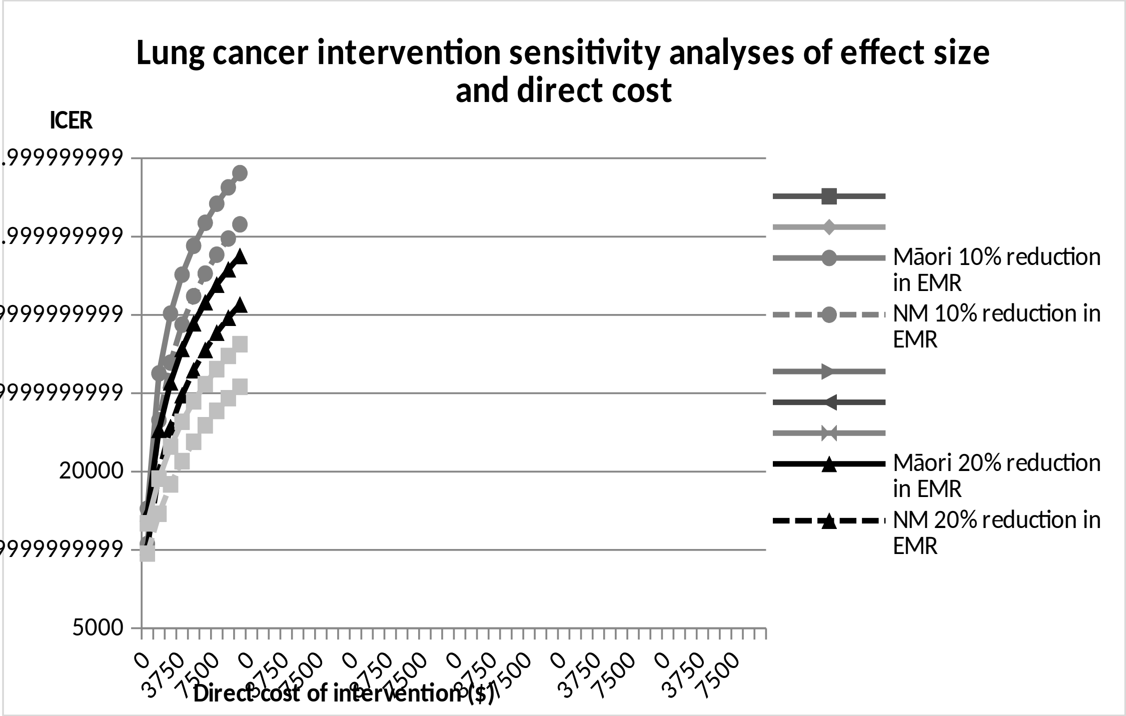
Lung cancer intervention sensitivity analyses of effect size and direct cost
ICER
319999.999999999
159999.999999999
79999.9999999999
39999.9999999999
20000
9999.9999999999
5000
0
3750
7500
Direct cost of intervention ($)
Māori 10% reduction in EMR
NM 10% reduction in EMR
Māori 20% reduction in EMR
NM 20% reduction in EMR

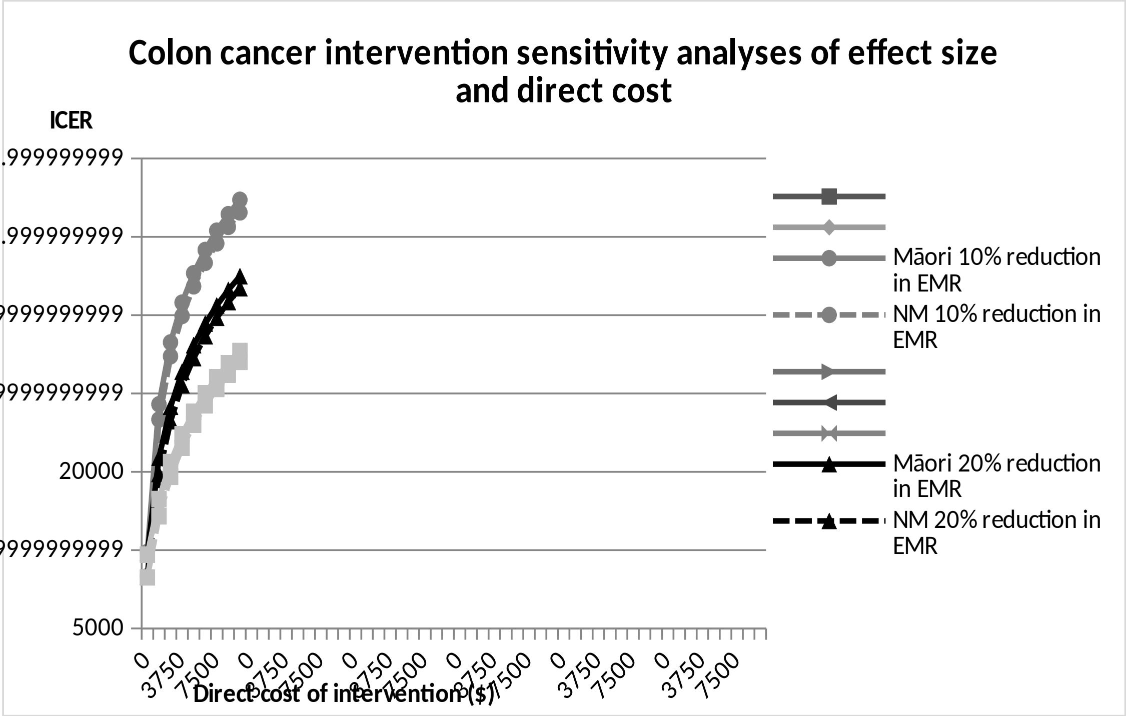
Colon cancer intervention sensitivity analyses of effect size and direct cost
ICER
319999.999999999
159999.999999999
79999.9999999999
39999.9999999999
20000
9999.9999999999
5000
0
3750
7500
Direct cost of intervention ($)
Māori 10% reduction in EMR
NM 10% reduction in EMR
Māori 20% reduction in EMR
NM 20% reduction in EMR

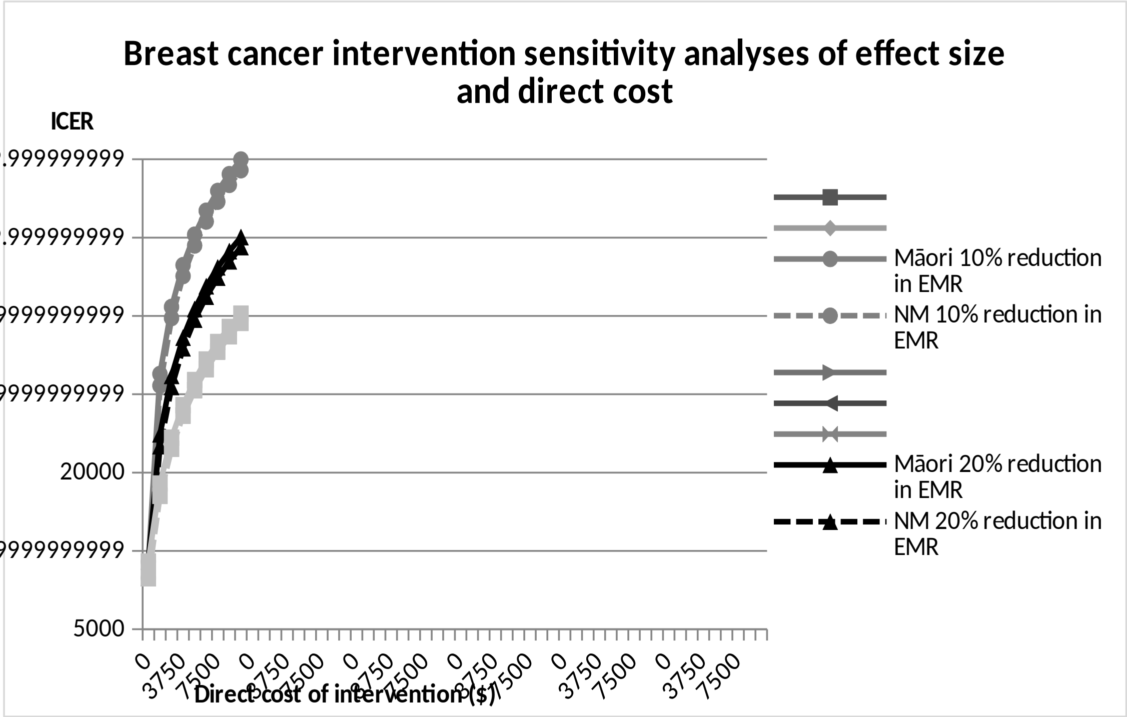
Breast cancer intervention sensitivity analyses of effect size and direct cost
ICER
319999.999999999
159999.999999999
79999.9999999999
39999.9999999999
20000
9999.99999999999
5000
0
3750
7500
0
3750
7500
0
3750
7500
0
3750
7500
0
3750
7500
0
3750
7500
Direct cost of intervention ($)
Māori 10% reduction in EMR
NM 10% reduction in EMR
Māori 20% reduction in EMR
NM 20% reduction in EMR
