# Supplementary material for: Why equal treatment is not always equitable: the impact of existing ethnic health inequalities in cost-effectiveness modeling
Source: Popul Health Metr. 2014 Jun 2;12:15. doi: 10.1186/1478-7954-12-15 (PMC4047777; doi:10.1186/1478-7954-12-15)
Supplement: Supplementary file 4 [file 1478-7954-12-15-S3.docx]

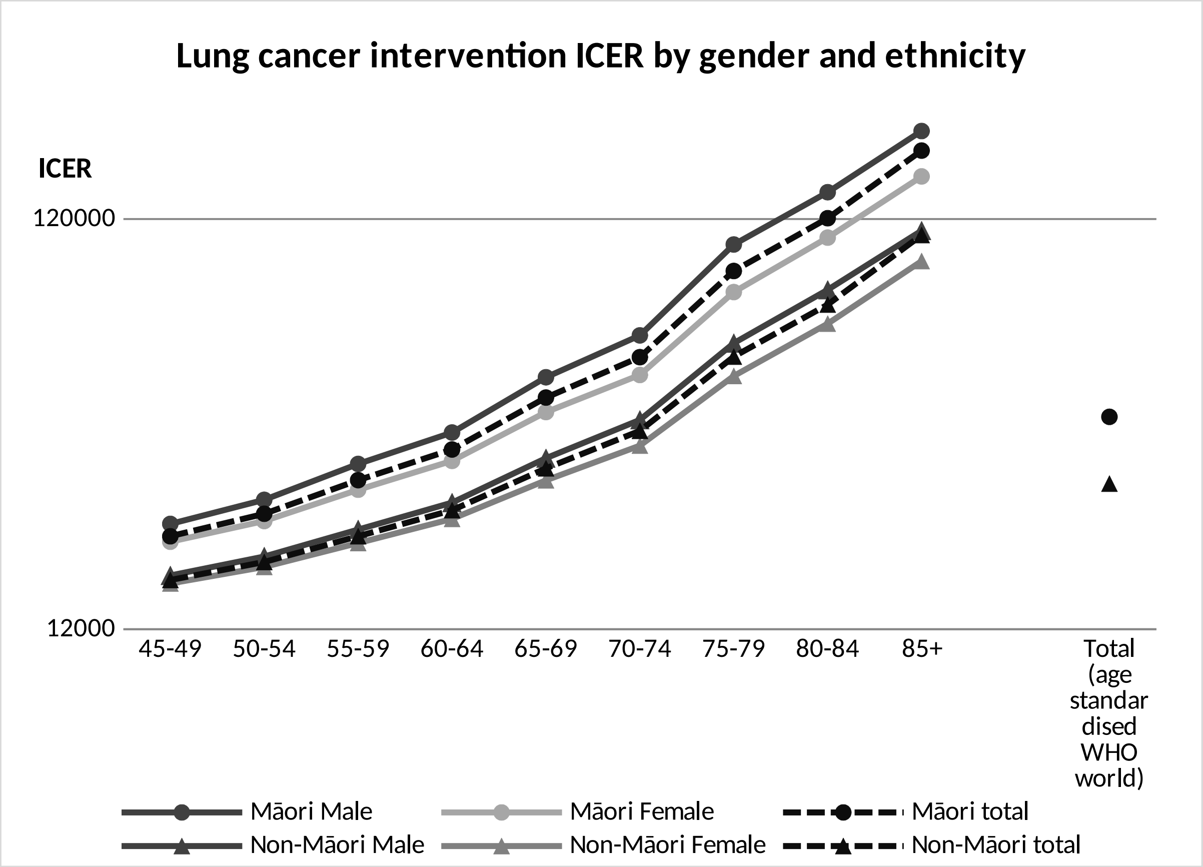

Lung cancer intervention ICER by gender and ethnicity
ICER
120000
12000
45-49
50-54
55-59
60-64
65-69
70-74
75-79
80-84
85+
Total (age standardised WHO world)
Māori Male
Māori Female
Māori total
Non-Māori Male
Non-Māori Female
Non-Māori total
